# Self-control and Task Timing Shift Self-efficacy and Influence Willingness to Engage in Effortful Tasks

**DOI:** 10.3389/fpsyg.2017.01788

**Published:** 2017-10-11

**Authors:** Danit Ein-Gar, Yael Steinhart

**Affiliations:** Marketing Department, Coller School of Management, Tel Aviv University, Tel Aviv, Israel

**Keywords:** self-control, self-efficacy beliefs, effortful tasks, task timing, precommitment

## Abstract

Self-efficacy constitutes a key factor that influences people's inclination to engage in effortful tasks. In this study, we focus on an interesting interplay between two prominent factors known to influence engagement in effortful tasks: the timing of the task (i.e., whether the task is scheduled to take place in the near or distant future) and individuals' levels of self-control. Across three studies, we show that these two factors have an interacting effect on self-efficacy. Low self-control (LSC) individuals report higher self-efficacy for distant-future effortful tasks than for near-future tasks, whereas high self-control (HSC) individuals report higher self-efficacy for near-future tasks than for distant future tasks. We further demonstrate how self-efficacy then molds individuals' willingness to engage in those effortful tasks. Given that a particular task may comprise effortful aspects alongside more enjoyable aspects, we show that the effects we observe emerge with regard to a task whose effortful aspects are salient and that the effects are eliminated when the enjoyable aspects of that same task are highlighted.

## Introduction

In numerous everyday situations, whether at work or at home, individuals need to perform tasks that require effort. In general, individuals do not enjoy performing these effortful tasks, regardless of the benefits they promise. Given that so many tasks in our everyday lives demand effort, understanding how to encourage people to commit to engaging in such tasks is of considerable theoretical and practical importance.

A key factor that influences individuals' willingness to engage in effortful tasks is the extent to which they feel capable of completing such tasks successfully (Bandura, 1977; Bandura and Schunk, 1981; Ozer and Bandura, 1990; Judge and Bono, 2001; Cassidy, 2015). A few meta-analyses reaffirmed the important role self-efficacy plays in a variety of everyday tasks (Moritz et al., 1988; Holden, 1991; Sadri and Robertson, 1993; Stajkovic and Luthans, 1998; Luszczynska et al., 2005). More specific to this research, studies that have looked at demanding tasks show that people are more inclined to complete them when they perceive themselves as being more competent to execute these tasks successfully (Eden and Kinnar, 1991; Olson et al., 1996; Stajkovic and Luthans, 1998; Lee and Mendlinger, 2011; Huang et al., 2016).

In particular, we focus on the role of perceptions of task competence related to a specific task, termed as situation-specific self-efficacy (SSE), a concept that is distinct from general self-efficacy (GSE; Brockner, 1988; Eden, 1988). The literature on SSE has attributed shifts in one's competence perception as dependent upon task characteristics, such as task difficulty and task demands (Pinder, 1984; Eden and Kinnar, 1991), task environment (Gist and Mitchell, 1992), task autonomy (Parker, 1998) or task familiarity (Kanfer and Ackerman, 1989; Gist and Mitchell, 1992). Other factors that influence competence perceptions refer to the specific characteristics of the individual performing the task, such as gender and ethnicity (Bong, 1999), personality (i.e., the Big 5 personality traits), intelligence (Judge et al., 2007), information processing (Matsou et al., 2015), and expertise (Bong, 1999; Judge et al., 2007).

In this research, we explore the interaction between two fundamental factors that drive shifts in SSE when faced with a difficult task. First, we address the effect of time on SSE by relying on the notion that SSE is a dynamic construct (Wood and Bandura, 1989) that differs when individuals face a near- or a distant-future task. Second, we consider dispositional self-control (Mischel, 1974; Mischel et al., 1989; O'Gorman and Baxter, 2002; Ein-Gar and Sagiv, 2014). Dispositional self-control is strongly related to the inclination to complete effortful tasks (Ferrari and Emmons, 1995; Sirois, 2004; Steel, 2007). This inclination was found to differ across time (Ein-Gar, 2015), and thus constitutes an important factor to consider.

We propose that the consideration of SSE alongside these two factors should yield a better understanding of individual behavior. More specifically, adding to past research that has shown differences in SSE at different points in time, we reveal that these differences may have opposite patterns depending on their dispositional self-control. For individuals who are lower on the self-control continuum, we predicted SSE would be higher when the effortful task will take place in the distant rather than the near future. Conversely, for individuals who are higher on the self-control, we proposed that SSE would be lower when a task is expected to occur in the distant compared to the near future. Importantly, we demonstrate how these changes in SSE influence intentions to engage in effortful tasks.

The paper is organized as follows: First, we review literature on willingness to engage in effortful tasks and discuss its relation to self-efficacy. We then discuss how dispositional self-control and a task's time of execution influences SSE. We tie past findings together and formulate our hypotheses. Finally, we test these hypotheses in a set of three experiments in which we present participants with a variety of effortful tasks from different domains and assess their willingness to engage in such tasks.

## Literature review

### Self-efficacy and willingness to engage in effortful tasks

Self-efficacy is defined as the belief in one's capacity to organize and execute the courses of action required to manage prospective situations (Bandura, 1997). It is often viewed as either a stable trait that an individual carries across situations at a relatively constant level (i.e., GSE), or as a situation-specific state that varies within the same person across situations (i.e., SSE; Sherer et al., 1982; Eden and Kinnar, 1991; Chen et al., 2001).

In this research, we focus on SSE when facing effortful tasks. SSE plays a central role in motivating individuals' behaviors and driving them to take part in effortful tasks. The more effortful the task, the less efficacious individuals feel, and the less likely they are to engage in the task (Eden and Kinnar, 1991; Eden and Aviram, 1993; Huang et al., 2016). For example, Lee and Mendlinger (2011) showed that students are reluctant to take online courses when they perceive them to be too difficult.

Past research has captured several aspects influencing individual SSE, which influence engagement in effortful tasks. These aspects can be attributed to both the task characteristics and to the characteristics of the individual performing the task. Task characteristics which were found to influence SSE were task difficulty (Pinder, 1984; Eden and Kinnar, 1991; Gist and Mitchell, 1992; Judge et al., 2007) and task environment including the presence of distractions (e.g., noise, interruptions), psychological or physical risk embedded in task setting, and task location (Gist and Mitchell, 1992). The presence of others while doing the task, which provides information about the “correct” performance strategy, may also influence self-efficacy perceptions (Bandura, 1988, p. 143). In addition, prior research has considered task familiarity as a task characteristic that may influence SSE perceptions (Kanfer and Ackerman, 1989; Gist and Mitchell, 1992). Influential individual characteristics can be qualities like gender and ethnicity (Bong, 1999). Other individual characteristics found to influence self-efficacy were information-processing style (i.e., autonomic responses; Matsou et al., 2015). Judge et al. (2007) found that personality traits such as conscientiousness, extraversion, and emotional stability influence SSE in the context of job-related performance. In this research, we focus on yet another task characteristic: task timing.

### Task timing and self-efficacy

Prior research has linked both GSE and SSE to task timing in several ways. One research avenue has shown that self-efficacy tends to be different at present and future points in time when it is associated with a goal. For instance, Bandura (1982) found that GSE is higher when individuals face proximate goals as compared to distant ones. Proximate goals provide immediate incentives and guides for action compared with distant goals, which are too far removed in time to be useful in effectively mobilizing effort. Bandura (1982) suggests that, in general, individuals may be more willing to engage in near-future tasks because such tasks are linked to near-future goals, compared with distant-future tasks related to distant-future goals. Another research avenue on task timing shows that linking past tasks to future tasks also influences self-efficacy, and the same was found for progress during a continuous task. As individuals successfully advance in the completion of their tasks, their SSE increases and motivates them to continue pursuing their goals and take on additional tasks (Schunk, 1982, 1983; Ellis et al., 2010). In the current study, we focus on individual time perspectives toward a task (rather than retrospective time focus or time during completion of the task), keeping goals constant in the sense that the goals associated with the task are the same in the near and distant future. We test whether SSE is different when considering a “stand alone” task due in the near or distant future.

Temporal construal theory (Trope and Liberman, 2010), as a prominent theory of time, may be considered in the context of SSE. This theory suggests that future events are construed more abstractly than near events (Trope and Liberman, 2010), and abstractness activates high level construal which leads to goal-driven, controlled behavior (Fujita et al., 2006; Fujita and Roberts, 2010; Fujita and Carnevale, 2012). Hence, according to construal level theory, individuals may be more willing to engage in effortful tasks when they consider them in the distant as opposed to the near future. However, the relationship between temporal construal and self-efficacy is not clear. On the one hand, at higher construal levels individuals focus more on the desirability than the feasibility of the task (Liberman and Trope, 1998). Accordingly, distant future tasks, may be perceived as demanding less effort and may increase individuals' SSE. On the other hand, distant future tasks are estimated to demand more time than near future tasks (Kanten, 2011), and as such, may decrease individuals' SSE.

In the present research, we argue that some individuals may experience higher SSE for near-future tasks over distant-future ones, while others may experience the opposite pattern of SSE, such that for distant-future tasks, their SSE will be higher than for near-future tasks. Interestingly, we suggest that these opposite patterns in SSE levels at different points in time depend on one's dispositional self-control.

### Self-control and self-efficacy

The individual difference we explore in the current research is self-control—namely, a person's propensity to resist temptations in order to achieve long-term goals. This propensity is driven by inherent individual properties (Mischel, 1974; Mischel et al., 1989; O'Gorman and Baxter, 2002; Tangney et al., 2004; Hagger, 2013; Ein-Gar and Sagiv, 2014), yet can also be influenced or induced by situational factors (Baumeister et al., 1998; Hagger et al., 2010; Hofmann et al., 2012).

While self-control refers to intentional regulated actions in an attempt to achieve future goals, self-efficacy refers to individuals' beliefs in their abilities to execute actions successfully. Thus, it is not surprising that people who have been generally successful in regulating their behaviors and attaining their goals also hold high beliefs about their general abilities, which is evident by past research showing that the relationship between GSE and self-control is positive and relatively strong (e.g., Luszczynska et al., 2005; Gottschling et al., 2016). However, the relationship between SSE and self-control has received less attention. While GSE and SSE are obviously related, they are not the same. SSE has been found to be a stronger and more accurate predictor of task performance than GSE (Bandura, 1986, 1997), and it has been found to be more susceptible to situational induction (Chen et al., 2001).

We propose that it is important to test the relationship between dispositional self-control and SSE because they are two distinct theoretical constructs that may influence task engagement. SSE refers to one's belief in his or her ability to successfully execute a specific task; whereas dispositional self-control is a personal tendency to overcome or yield to temptations in general. As such, dispositional self-control reflects who people are (or what people think they are) rather than how well they expect to perform a specific task.

These two constructs are linked. According to Bandura's social cognitive theory, they are both key processes that affect individual behavior. As such, if an individual perceives him- or herself to be able to overcome temptations and accomplish goals frequently (i.e., be high in self-control) *and* believes s/he has the ability to successfully perform a specific demanding task, then s/he is likely to be highly motivated to engage in that particular task. However, individuals can hold opposing perceptions of self-control and SSE. One can perceive him-/herself as being high in self-control in general, and at the same time, believe s/he has little ability to perform a certain task adequately, and vice versa. Alternatively, an individual can perceive him-/herself as someone who tends to yield to temptations, yet feel very capable of completing a certain task (even if the task demands effort).

Therefore, in the current research, we investigate the assumed straightforward positive relations between self-control and self-efficacy, and explore the dynamic of these relations within the context of near- and distant-future tasks.

## The present research

**Figure 1:**
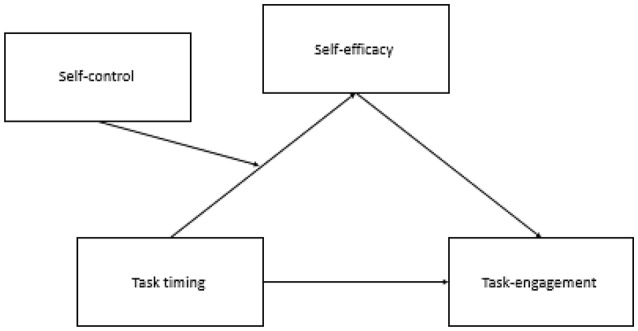
Self-efficacy mediates the effect of task timing (near future or distant future) on task engagement for different levels of self-control.

We propose that an individual's willingness to engage in effortful tasks depends on the relationships between three factors: the task's execution timing (i.e., near or distant future), the extent to which the individual believes in his or her ability to execute the task (i.e., SSE), and the individual's dispositional self-control (see Figure 1 for illustration). Individuals can be placed on different levels of the self-control spectrum. We propose that individuals who are placed at lower levels of the self-control spectrum (we term “LSC”) will experience different SSE at different points in time, in comparison to those who are placed at higher levels of the spectrum (we term “HSC”). Specifically, we propose that LSC individuals experience higher task efficacy when effortful tasks will occur in the distant future than when they will occur in the near future, whereas HSC individuals experience the opposite, with higher SSE for near over distant future tasks. These differences in SSE at different points in time influence LSC and HSC individuals' willingness to engage in the effortful tasks.

Individuals with LSC tend to postpone effortful tasks more than individuals with HSC (Ferrari and Emmons, 1995; Sirois, 2004; Steel, 2007). In fact, some scholars even perceive procrastination to be a subcomponent of self-control (e.g., Ferrari, 2001; Ein-Gar and Sagiv, 2014). Low self-efficacy was found to be associated with procrastination, especially within the domain of academic procrastination (e.g., Tuckman, 1991; Ferrari et al., 1992; Haycock et al., 1998; Wolters, 2003; Steel, 2007; Klassen et al., 2008). Sirois (2004) has found that procrastination is associated with both LSC and low self-efficacy. Because that study reported a correlational relationship without causality, it could be argued that individuals with either LSC or low self-efficacy (or both) would express procrastination and be less willing to engage in effortful tasks in the near future. However, there is some new evidence showing that LSC individuals, under certain circumstances, may be more willing to expedite, rather than procrastinate, engagement in effortful tasks and HSC individuals may demonstrate the opposite behavior (Ein-Gar, 2015, Study 4), hinting that perhaps self-control is not the direct force driving future intentions for behavior. In that study, both HSC and LSC individuals were more willing to pre-commit to a task when they anticipated having more available time to uphold their future commitment. However, their time availability estimation was dependent on how they construed their future schedule.

In this research, we suggest that both HSC and LSC individuals express a certain “planning fallacy” (Buehler et al., 1994, 2010). This fallacy occurs because distant future situations are interpreted differently than near future situations. We argue that HSC and LSC individuals have different interpretations and expectations regarding distant vs. near future effortful tasks, and these are manifested in their task efficacy perceptions.

LSC individuals may be more likely to engage in effortful tasks in the distant than in the near future not only because of their tendency to avoid or delay effortful tasks, but also because they experience lower SSE in the near than in the distant future. When it comes to deciding whether to engage in a future effortful task or not, LSC individuals' “planning fallacy” (Buehler et al., 2010) expresses an optimistic-bias toward future events. Optimism bias in the context of planning ahead tasks suggests that individuals are more optimistic about successfully completing tasks, and about how long those tasks would take, for distant future tasks compared with near future tasks (Buehler et al., 1994; Buehler and Griffin, 2003). Past research provides initial evidence for this bias in showing that LSC individuals assume they will have more time in the distant future than in the near future for a pre-committed task (Ein-Gar, 2015). In this sense, LSC individuals are not naïve because they think they will be more proficient in the future, but because they neglect to anticipate future situational obstacles that will prevent them from completing the task (O'Donoghue and Rabin, 1999). As such, LSC individuals' efficacy is driven by their optimistic assessment that they will be more able to uphold their commitment in the distant future over to the near future. Consequently, we predict that LSC individuals feel greater SSE with regard to effortful distant-future than near-future tasks.

HSC individuals, however, do not interpret future situations in the same way as LSC individuals. As such, although they also express a “planning fallacy,” unlike LSC individuals, their fallacy signals more pessimism about the future. We suggest that, compared with “optimism-biased” LSC individuals, HSC individuals who are highly responsible and conscientious (O'Gorman and Baxter, 2002; Tangney et al., 2004; Olson, 2005) may hold more pessimistic perceptions regarding their ability to carry out future tasks. Pessimism regarding future events is strongly related to responsibility related concerns and worries. The more individuals are concerned with possible future negative outcomes, the more they believe in the chances these outcomes will occur (MacLeod et al., 1991). As such, HSC individuals may be more concerned with being able to uphold their commitments and complete the tasks. These concerns are heightened when forecasting distant future events, which hold ambiguity (MacLeod et al., 1991). In such cases, HSC individuals may feel more doubtful about whether the circumstances in the distant future would enable them to uphold their commitment. Initial evidence shows that HSC participants believe they will have more time to uphold a pre-commitment when it is due in the near future than in the distant future (Ein-Gar, 2015). In this vein, SSE is proposed to be driven by HSC individuals' pessimistic interpretations of the future events and the circumstances that will enable the adequate completion of the task. Accordingly, we suggest that HSC individuals feel greater SSE for tasks that are proximate to their current state than for distant-future tasks.

Importantly, our prediction that HSC individuals may experience different levels of self-efficacy at different points in time is not a trivial one. Research has shown that positive task experience increases SSE. This effect was found among children (e.g., Schunk, 1982, 1983), as well as adults (e.g., Ellis et al., 2010). Therefore, it could be assumed that HSC individuals who experienced success in past effortful tasks would have higher SSE for similar tasks, thus encouraging their engagement with them, regardless of the task's timing. In this research, we counter this intuition by arguing that HSC individuals do experience different levels of SSE at different points in time, even if the tasks at hand are common tasks that they might have executed successfully in the past.

In sum, LSC and HSC individuals interpret the situational circumstances of effortful tasks differently at different time points, and this is expressed in their perception that they can complete these tasks successfully. Therefore, it is not that they perceive the task to differ in terms of the effort it demands, but rather they derive their SSE from the situational circumstances, such as their expected time availability. More formally, we predict that LSC individuals are more willing to engage in an effortful task when it is due in the distant future rather the near future, whereas HSC individuals are more likely to engage in the task if it is due in the near as opposed to the distant future. The effect is mediated by SSE for both LSC and HSC individuals.

## Materials and methods

We explore our hypotheses in three experiments, testing different types of effortful tasks. All measures, manipulations, and exclusions in these experiments are disclosed. All studies were carried out in accordance with the recommendations of the Ethics committee of Tel-Aviv University. All subjects gave written informed consent online in accordance with the Declaration of Helsinki.

## Experiment 1: joining a financial coaching program

In experiment 1, participants considered an offer to join a free personal financial coaching program that focuses on a responsible financial lifestyle. We expected to replicate past findings by showing that HSC participants indicate greater willingness to join a program that is scheduled to take place in the near future rather than to join a program scheduled for the distant future, whereas LSC participants indicate greater willingness to join a distant-future program. Extending past findings, we expect this effect to be driven by self-efficacy. All measures, manipulations, and exclusions in this experiment and the experiments that follow are included.

### Methods

#### Participants

In this study, 259 participants (*M*_age_ = 30, 40% women) completed the experiment using the Amazon Mechanical Turk (mTurk) survey platform in exchange for $1.00 (USD). In this study and all subsequent studies, we targeted a sample size with 80% power to detect a moderate effect (*f* ^2^ = 0.25).

#### Procedure and measures

First, participants completed the Dispositional Self-Control (DSC) scale (Ein-Gar and Steinhart, 2011; Ein-Gar and Sagiv, 2014). This scale consists of 17 items (each measured on a 5-point scale, α = 0.89) measuring participants' tendencies to overcome temptations (e.g., “usually, when something tempts me, I manage to withstand it”) or yield to them (e.g., “I often make spontaneous and rather hasty decisions”). Next, participants read about a hypothetical coaching program offered by a bank, free of charge to current and potential clients. The purpose of the program was to train people to implement a financially responsible lifestyle (Appendix A in Supplementary Material provides a full description of the scenario). The results of a pretest (*n* = 35, *M*_age_ = 33.35, 46% women) confirmed that individuals perceived the program as effortful (*M* = 5.43, *SD* = 1.45) and important (*M* = 5.20, *SD* = 1.58), compared to a mid-point of 4 [*t*_(34)_ = 7.38, *p* < 0.005; *t*_(34)_ = 4.47, *p* < 0.005, respectively], but not enjoyable [*M* = 2.80, *SD* = 1.37; *t*_(34)_ = −4.89, *p* < 0.005]. These perceptions were not affected by self-control levels (*r* < 0.26, *p* > 0.13).

Each participant was randomly assigned to one of two task-timing conditions (near-future or distant-future condition). We informed participants that the program would begin either next month (near-future condition) or in 3 months (distant-future condition).

Pre-test participants (*n* = 121, *M*_age_ = 38.21; 47% women) were randomly assigned to one of the time conditions. They read the scenario and used a 7-point scale to report to what extent would they consider the tasks to take place in the near or distant future, how far in time the tasks seem, and how close or distant in time they perceive the stating date of the tasks (α = 0.94). Participants in the 1 month condition perceived the tasks as closer in time (*M* = 2.63, *SD* = 1.32) than participants in the 3 months condition [*M* = 4.01, *SD* = 1.37; *t*_(119)_ = 5.63; *p* < 0.001].

After reading the scenario, we asked participants to indicate their willingness to join the coaching program on a 7-point scale (1 = “not at all,” 7 = “very much”). Finally, participants estimated their self-efficacy by rating how confident they were in their ability to (a) prepare all the relevant documents for the meetings, (b) comprehend the suggestions and tools offered in the coaching sessions, and (c) maintain the new financial guidelines as a way of life. Participants responded to each of these items using a 100-point scroll bar (α = 0.88).

### Results and discussion

#### Self-efficacy

We conducted a regression analysis where self-control, task timing (0 = near-future, 1 = distant-future), and the interaction between the two served as predictors of participants' self-efficacy. Table 1 provides descriptive statistics and correlation analyses. We observed significant effects for self-control, *b* = 17.64, *SE* = 3.36; *t*_(257)_ = 5.25, *p* < 0.001, and for task timing, *b* = 46.81, *SE* = 16.59; *t*_(257)_ = 2.82, *p* = 0.0052. Importantly, the interaction between the two was significant, *b* = −13.23, *SE* = 4.61; *t*_(257)_ = −2.87, *p* = 0.045, *R*^2^ = 0.104.

**Table 1:** Experiment 1.

	**Task timing**	**Self-control**	**Self-efficacy**	**Willingness to join the program**
Mean	0.50	3.55	72.10	3.48
*SD*	0.50	0.59	23.02	1.85
Task timing		−0.001	−0.003	−0.05
Self-control			0.27^**^	−0.07
Self-efficacy				0.27^**^

** *p < 0.001*.

**Figure 2:**
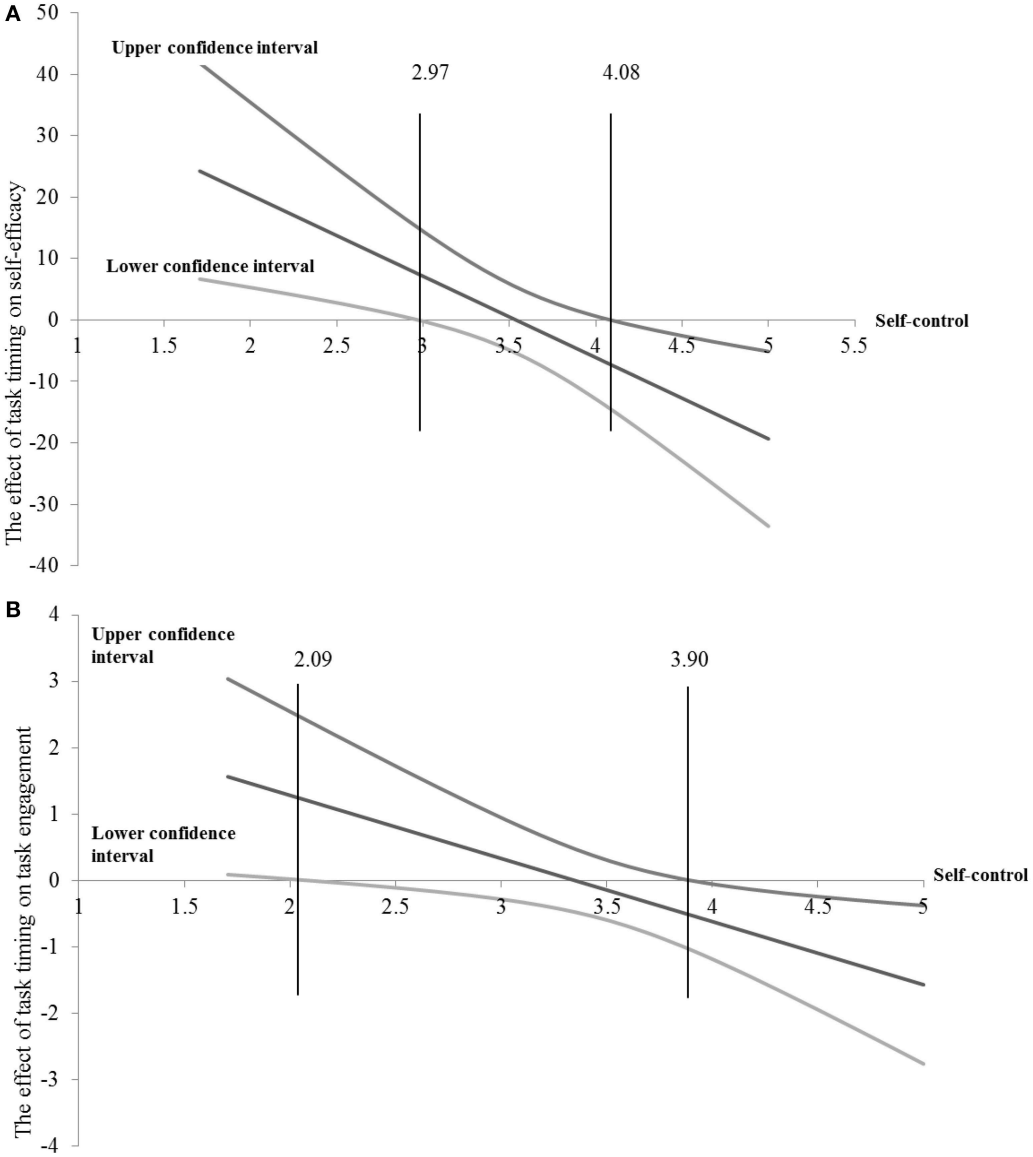
**(A)** Effect of task timing on self-efficacy as a function of self-control levels. The graph was drawn on the basis of a floodlight analysis (Spiller et al., 2013; Disatnik and Steinhart, 2015), which examines the effect of task timing on self-efficacy for any value that self-control can take. Confidence bands are also presented, and the Johnson–Neyman points are obtained at self-control = 2.97 and self-control = 4.08 (*p* = 0.05). **(B)** Effect of task timing on willingness to join a financial coaching program as a function of self-control levels. The graph is based on a floodlight analysis (Spiller et al., 2013; Disatnik and Steinhart, 2015), which examines the effect of task timing on willingness to join a financial coaching program for any value that self-control can take. Confidence bands are also presented, and the Johnson–Neyman points are obtained at self-control = 2.09 and self-control = 3.90 (*p* = 0.05).

We decomposed the interaction using the Johnson-Neyman “floodlight” approach recommended by Spiller et al. (2013; see also Disatnik and Steinhart, 2015). Recent research has recommended using this approach in cases where there are no focal values (McClelland et al., 2015; Krishna, 2016). “Floodlight” analysis enables one to report regions of the continuous X variable where the simple effect of the manipulated Z is significant (Spiller et al., 2013). In the current research, the floodlight analysis demonstrates the regions of self-control (i.e., continuous X variable) under which the manipulated effect of task timing (i.e., manipulated Z) is significant. As depicted in Figure 2A, task timing had a significant positive effect on self-efficacy for self-control levels lower than 2.97 (*b*_JN_ = 7.51, *SE* = 3.81, *p* = 0.05) and a significant negative effect on self-efficacy for self-control levels higher than 4.08 (*b*_JN_ = −7.24, *SE* = 3.68, *p* = 0.05).

#### Task engagement

We conducted a second regression on willingness to join the coaching program. The effect of task timing, *b* = 3.19, *SE* = 1.39; *t*_(257)_ = 2.29, *p* = 0.0227, was significant, as was the interaction between the two, *b* = −0.95, *SE* = 0.39; *t*_(257)_ = −2.46, *p* = 0.0145, *R*^2^ = 0.031. As presented in Figure 2B, task timing had a significant positive effect on willingness to enroll in the coaching program when self-control levels were lower than 2.09 (*b*_JN_ = 1.19, *SE* = 0.61, *p* = 0.05), and a significant negative effect when self-control levels were higher than 3.90 (*b*_JN_ = −0.52, *SE* = 0.27, *p* = 0.05).

#### Underlying process

We conducted a moderated mediation analysis, using bootstrapping mediation tests (Hayes, 2013) with 5,000 replications. In Hayes's Model 7, self-control served as the moderator for the effect of task timing on willingness to join the coaching program, and self-efficacy was the mediator. For both HSC and LSC participants, the effect of task timing was mediated by self-efficacy: For HSC participants (1 *SD* above the mean), self-efficacy and, consequently, their decision to join the program were higher for the near-future condition than for the distant-future condition (*b* = −0.17, *SE* = 0.09; 95% CI: −0.40 to −0.03). For LSC participants (1 *SD* below the mean), self-efficacy and, subsequently, their willingness to join the program were higher for the distant-future condition than for the near-future condition (*b* = 0.17, *SE* = 0.09; 95% CI: 0.01−0.39). Thus, this experiment demonstrated the role self-efficacy plays in determining willingness to engage in an effortful task across different levels of self-control.

One alternative explanation, which could account for the effect found in Experiment 1, is that participants perceived the task to be less effortful in the distant future and, hence, expected it to demand less self-control. According to this explanation, LSC individuals will be more willing to engage in the task in the distant future because it seems less effortful. This will also influence their self-perceptions regarding their ability to perform the task. Although, this explanation does not account for HSC individuals' preferences to engage in the task in the near future over the distant future, it is important to rule this out as an explanation for LSC individuals' behaviors. To that end, in the next study, we also test whether or not task effort perceptions are different in the near compared with the distant future.

## Experiment 2: joining a running/walking group

The goal of experiment 2 was to reproduce the findings of experiment 1 with a different, somewhat enjoyable effortful task to emphasize that effort is not only associated with undesirable tasks, and to rule out the alternative explanation that perceptions of effort are different at different points in time. Moreover, participants were led to believe that their decisions would be real and binding.

In this experiment, we primed self-control instead of measuring it. There are two types of self-control manipulations. One type manipulates the actual state of an individual's self-control resources, such that after performing the task, participants are in an ego-depleted state and do not have sufficient resources to adequately complete subsequent tasks that demand self-control (Baumeister et al., 1998; Hagger et al., 2010). Therefore, when manipulating actual resources, depleted participants are not expected to be enthusiastic to engage in future effortful tasks whether they are expected in the near or distant future. Non-depleted participants (i.e., those who did not complete a depleting task), however, are expected to show a crossover effect where an individual's engagement in the effortful task depends upon task timing and their dispositional self-control level. The other type of manipulation does not change an individual's resource state but rather their perception of who they are. That is, whether they think they tend to behave in a manner that demonstrates high or low self-control (LSC). In this study, we employed the second type of manipulation, showing the moderating effect of perceived self-control rather than state self-control.

### Methods

#### Participants

In this study, 108 participants (*M*_age_ = 31.7, 48% women) completed an online survey in exchange for $3.00.

Participants in Study 2 were approached via an online survey database website. This website serves as an online platform for studies with a representative subject pool of more than 30,000 users representing a broad range of demographics (e.g., gender, age, education, income etc.). Participants anonymously complete studies and surveys in return for monetary compensation.

#### Procedure and measures

Participants were each randomly assigned to one of four conditions in a 2 (self-control priming: HSC or LSC) × 2 (task timing condition: near-future or distant-future) between-subjects design.

First, participants underwent a self-control priming manipulation. Since this paper focuses on dispositional self-control rather than state self-control, we used a manipulation that influences a participant's perception of who they are in terms of self-control as opposed to manipulating their actual self-control resources, as in the case of ego-depletion manipulations. In the HSC condition, participants were instructed: “Recall an experience in which you overcame an urge or a temptation. Please describe the experience in detail, including the events that took place” In the LSC condition, participants were instructed: “Recall an experience in which you yielded to an urge or a temptation. Please describe the experience in detail, including the events that took place” (Adopted from: vanDellen and Hoyle, 2010; Ein-Gar and Steinhart, 2011). This manipulation was pre-tested with participants from the same sample pool as study 2 (*n* = 89, *M*_age_ = 30.34, 51.7% women). Participants completed one of two versions of the recall task and the DSC scale. As expected, participants in the overcoming temptations condition reported higher self-control perceptions (*M* = 3.53, *SD* = 0.53) than participants in the yielding to temptations condition [*M* = 3.29, *SD* = 0.61, *t*_(87)_ = 1.98; *p* = 0.05].

Next, participants read about an offer to join a running/walking group with a professional trainer (see Appendix A in Supplementary Material). An initial pretest (*n* = 55, *M*_age_ = 31.47, 40% women) confirmed, that individuals perceived this task as effortful (*M* = 4.89, *SD* = 1.51) and important (*M* = 4.82, *SD* = 1.60), compared to scale a mid-point of 4 [*t*_(54)_ = 4.37, *p* < 0.005; *t*_(54)_ = 3.79, *p* < 0.005, respectively]. Participants rated the task as somewhat enjoyable [*M* = 4.56, *SD* = 1.42; *t*_(54)_ = 2.43, *p* < 0.05]. These ratings were not affected by self-control levels (*r* < 0.07, *p* > 0.61).

Participants were told that the training program would begin either next month (near-future condition) or in 4 months (distant-future condition). We asked participants to indicate their willingness to join the program by stating the number of sessions they would be willing to sign up for, knowing that each session would cost a discounted rate of $5.00 instead of the full price of $10.00. We informed participants that once they indicated the number of sessions they wanted they would be transferred to a secure webpage where they would pay for those sessions. This was done so that participants would think they were actually joining the program and would report their sincere intentions.

Pre-test participants (*n* = 112, *M*_age_ = 37.47, 43% women) randomly assigned to one of the time conditions read the scenario and reported their time perception of the tasks on a 7-point scale using the 3 questions from Study 1 (α = 0.96). Participants in the 1 month condition perceived the tasks as being closer in time (*M* = 2.48, *SD* = 1.36) than participants in the 4 months condition [*M* = 3.37, *SD* = 1.25; *t*_(110)_ = 7.64; *p* < 0.001].

Next, participants reported on a 7-point scale (1 = “not at all” to 7 = “very much”) how much they believed they (a) would consistently attend the running/walking group, (b) would be able to uphold their commitment to participate on a regular basis, and (c) possess the ability to successfully persist in the program. The average score across all items served as the self-efficacy measure (α = 0.80). To test participants perceptions of task effort, we asked them to rate the extent to which the task demanded effort on a 7-point scale (1 = “not at all” to 7 = “very much”). Finally, we debriefed participants, informed them that the training program was hypothetical, and gave them an additional payment as compensation.

### Results and discussion

#### Effort perceptions

We conducted a regression analysis where self-control (0 = LSC, 1 = HSC), task timing (0 = near-future, 1 = distant-future), and the interaction between them served as the predictors, and effort perceptions served as the predicted variable. While effort perceptions were significantly above the scale mid-point and replicated the results of the task pre-test [*M* = 4.81, *t*_(108)_ = 4.99; *p* < 0.001], none of the effects in the regression were found to be significant. Specifically, the effect of self-control [*b* = −1.11, *t*_(106)_ = 1.14, *p* = 0.26], the effect of task timing [*b* = −0.86, *t*_(106)_ = −0.80, *p* = 0.42], and the interaction effect [*b* = 0.53, *t*_(106)_ = 0.79, *p* = 0.43] were not significant. These results indicate that effort perceptions do not differ at different points in time based on different levels of self-control.

#### Self-efficacy

**Figure 3:**
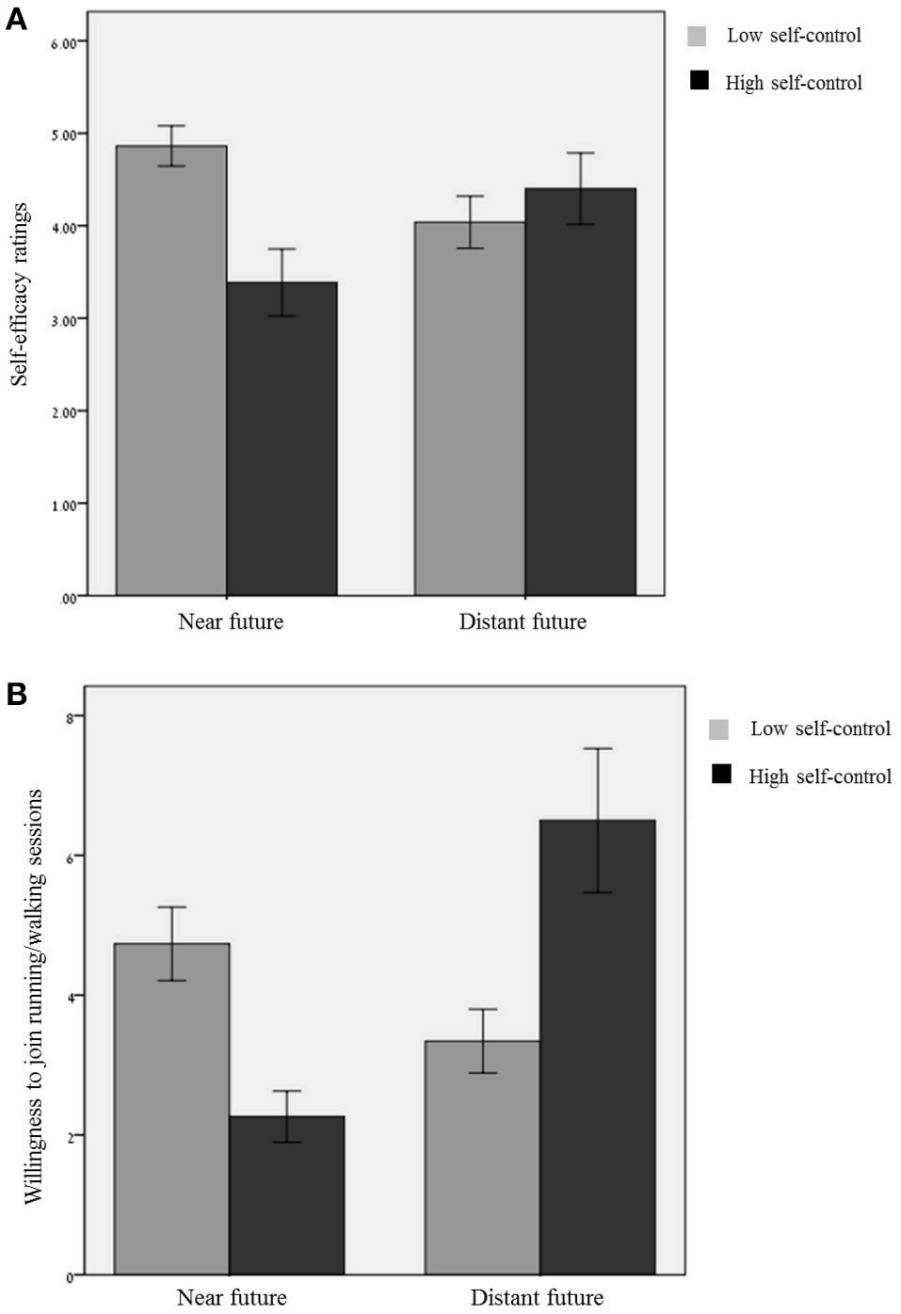
**(A)** Effect of task timing on self-efficacy as a function of self-control levels. **(B)** Effect of task timing on willingness to join a running/walking group as a function of self-control levels.

We conducted an additional regression analysis where self-efficacy served as the predicted variable (Table 2 provides descriptive statistics and correlation analyses). The effects of self-control, *b* = 1.48, *SE* = 0.44; *t*_(106)_ = 3.32, *p* = 0.001, and task timing, *b* = 1.01, *SE* = 0.49; *t*_(106)_ = 2.04, *p* = 0.044, were both significant. Importantly, the interaction between the two factors was significant, *b* = −1.84, *SE* = 0.62; *t*_(106)_ = −2.96, *p* = 0.0038; η^2^ = 0.078. As predicted, participants in the LSC condition reported higher self-efficacy when the program start date was presented in the distant future (*M* = 4.40, *SD* = 1.73) rather than in the near future [*M* = 3.38, *SD* = 1.58; *t*_(106)_ = 2.04, *p* = 0.044]. In contrast, participants in the HSC condition reported higher self-efficacy when the program start date was presented in the near future (*M* = 4.86, *SD* = 1.27) rather than in the distant future [*M* = 4.04, *SD* = 1.67; *t*_(106)_ = −2.20, *p* = 0.029; see Figure 3A].

**Table 2:** Experiment 2.

	**Task timing**	**Self-control**	**Self-efficacy**	**Number of sessions**
Mean	0.47	0.63	4.25	4.18
*SD*	0.50	0.48	1.61	3.37
Task timing		−0.03	−0.05	0.09
Self-control			0.16	−0.06
Self-efficacy				0.52^**^

** *p < 0.001*.

#### Task engagement

An additional regression analysis including the number of sessions that participants signed up for revealed significant effects for task timing, *b* = 4.24, *SE* = 0.99; *t*_(106)_ = 4.25, *p* < 0.0001, and self-control condition, *b* = 2.47, *SE* = 0.89; *t*_(106)_ = 2.77, *p* = 0.007. Importantly, the interaction between the two was significant, *b* = −5.62, *SE* = 1.24; *t*_(106)_ = −4.51, *p* < 0.001; η^2^ = 0.164, such that participants in the LSC condition were willing to sign up for more sessions when the program start date was presented in the distant future (*M* = 6.50, *SD* = 4.5) rather than in the near future [*M* = 2.26, *SD* = 1.59; *t*_(106)_ = 4.25, *p* < 0.001]. In contrast, participants in the HSC condition were willing to sign up for more sessions when the program start date was presented in the near future (*M* = 4.74, *SD* = 3.07) rather than in the distant future [*M* = 3.34, *SD* = 2.70; *t*_(106)_ = −1.86, *p* = 0.06; see Figure 3B].

#### Underlying process

We conducted a moderated mediation analysis using bootstrapping mediation tests (Hayes, 2013) with 5,000 replications. In Hayes's Model 7, self-control served as the moderator for the effect of task timing on willingness to sign up for sessions, and self-efficacy was the mediator. The effect of task timing was mediated by self-efficacy for both self-control conditions: For HSC participants, self-efficacy was higher for the near-future condition than for the distant-future condition and, consequently, determined participants' willingness to sign up for sessions (*b* = −0.91, *SE* = 0.44; 95% CI: −1.88 to −0.15). For LSC participants, self-efficacy was higher for the distant-future condition than for the near-future condition, which ultimately influenced the number of sessions participants signed up for (*b* = 1.11, *SE* = 0.63; 95% CI: 0.00–2.45).

Experiment 2 reproduced the results of experiment 1 in a setting with the following conditions: (a) self-control was induced rather than measured, (b) the focal task was a bit more enjoyable, (c) there was some financial burden associated with committing to the task, and (d) participants were led to believe they were making a binding commitment.

Furthermore, in this experiment, we demonstrated that the extent to which the task was perceived as demanding effort did not differ across levels of self-control and across time; this ruled out the possibility that the effect was driven by perceiving the distant-future task as less effortful and thus less demanding of self-control than the near-future task. Experiments 1 and 2 demonstrated this effect for tasks that all participants perceived as effortful. However, in some cases, a task may be perceived as effortful by some, but not by others. As a result, we might see differences in the magnitude of the effect. Therefore, it is essential to test whether shifts in task effort perception can alter the effect, thus demonstrating its malleability.

## Experiment 3: planning a vacation

The final experiment investigated whether the perception that a task is effortful is a necessary component in the mediating role of self-efficacy on individual willingness to engage in that task. We suggest that the relationships we have observed thus far between self-control and task timing in willingness to engage in a task, as well as the mediating role of self-efficacy, emerge only for tasks that individuals perceive as effortful. To investigate this hypothesis, we asked participants to consider planning a vacation while directing their focus to either the enjoyable or effortful aspects of this task.

### Methods

#### Participants

In this study, 196 participants (*M*_age_ = 34, 38% women) completed the experiment using the mTurk survey platform in exchange for $1.00.

#### Procedure and measures

First, participants completed the DSC scale (α = 0.92) used in experiment 1. Next, each participant was randomly assigned to one of four conditions in a 2 (task focus: effort-focused or enjoyment-focused) × 2 (task timing: near-future or distant-future) between-subjects design. Participants were asked to assume that they were planning a vacation. In the effort-focused condition, participants read: “You expect that preparing this vacation will demand time and effort.” In the enjoyment-focused condition, participants read: “You expect that preparing this vacation will be pleasant and fun.” Participants were informed that their travel agent had found a great deal for the flight, that the deal would expire in 1 month (the end of January, near-future condition) or within 6 months (the end of June, distant-future condition), and that they needed to register for the deal in advance to get the early-bird discount (see Appendix A in Supplementary Material). A pretest (*n* = 50, *M*_age_ = 33.44, 44% women) confirmed, based on ratings on a 7-point scale, that individuals perceived the task in the effort-focused condition as more effortful (*M* = 5.33, *SD* = 1.09), more demanding (*M* = 5.00, *SD* = 1.2), and more of a hassle (*M* = 4.97, *SD* = 1.67) compared to the task in the enjoyment-focused condition (*M* = 4.45, *SD* = 1.5; *M* = 3.5, *SD* = 1.85; *M* = 2.9, *SD* = 2.02, respectively). The differences in effort perception were significant *t*_(48)_ = −2.41, −3.48, −3.94, respectively; all *p* < 0.02) The task in the effort-focused condition was also perceived as less enjoyable (*M* = 2.90, *SD* = 1.13), less fun (*M* = 3.13, *SD* = 1.46), and less pleasurable (*M* = 2.93, *SD* = 1.41) compared with the task in the enjoyment-focused condition (*M* = 5.20, *SD* = 1.61; *M* = 5.10, *SD* = 1.89; *M* = 4.90, *SD* = 1.59, respectively). The differences in effort perception were significant *t*_(48)_ = 5.96, 4.15, 4.59, respectively; all *p* < 0.001.

In another pretest, participants (*n* = 119, 44% women, *M*_age_ = 36.27) were randomly assigned to one of the time conditions, read the scenario and reported their time perceptions of the task on a 7-point scale for the same 3 questions from Study 1 pre-test (α = 0.95). Participants in the 1 month condition perceived the tasks as being closer in time (*M* = 2.29, *SD* = 1.43) than participants in the 6 months condition [*M* = 3.94, *SD* = 1.47; *t*_(117)_ = 6.21; *p* < 0.001].

Next, participants reported their interest in signing up for the deal now and obligating themselves to organize the summer vacation in advance. They answered the question: “How interested are you in booking the flight now (to reserve seats and get a good price), thus obligating yourself to preparing the vacation by January/June?” on a 7-point scale ranging from 1 (not at all) to 7 (very willing). Then, participants answered the following question: “How concerned are you that you will not be able to arrange the vacation properly?” on a 7-point scale ranging from 1 (not concerned at all) to 7 (very concerned). The latter question served as the self-efficacy measure (reverse coded).

### Results and discussion

#### Self-efficacy

We conducted a regression analysis where task timing (1 = near-future condition, 2 = distant-future condition), self-control, task focus (0 = enjoyment-focused, 1 = effort-focused), all the two-way interactions between the independent variables, and the three-way interaction between them served as the predictors, and self-efficacy perceptions served as the predicted variable (using the PROCESS bootstrapping method, Model 3, with 5,000 replications; (Hayes, 2013). Table 3 provides descriptive statistics and correlation analyses. The full results are presented in Table 4, column 1.

**Table 3:** Experiment 3.

	**Task timing**	**Self-control**	**Task focus**	**Self-efficacy**	**Willingness to plan a vacation**
Mean	0.47	3.50	0.64	5.09	5.72
*SD*	0.50	0.68	0.48	1.54	1.29
Task timing		0.008	−0.05	0.09	−0.001
Self-control			−0.16^*^	0.32^**^	0.18^*^
Task focus				−0.07	−0.14
Self-efficacy					0.31^**^

* p < 0.05,

** *p < 0.001*.

**Table 4:** Self-efficacy and willingness to plan a vacation, as a function of self-control, task timing, and task focus (Experiment 3).

	**Self-efficacy**	**Willingness to plan the vacation**
Constant	5.06 (1.04)	4.92 (1.19)
Time of task performance	0.16 (0.21)	−1.64 (1.63)
Self-control	0.66^**^ (0.15)	0.33 (0.32)
Task focus	−0.06 (0.22)	−1.93 (3.08)
Task timing × self-control	−0.78^*^ (0.31)	0.34 (0.44)
Task timing × task focus	0.06 (0.44)	6.15^**^ (1.99)
Self-control × task focus	−0.40 (0.32)	0.40^*^ (0.39)
Task timing × self-control × task focus	−1.55^*^ (0.65)	−1.62^**^ (0.55)
R Square	0.17	0.13
Overall *F*	5.38	4.06
*df*	7,188	7,188

In the two regressions reported in the table, the predictors were as follows: self-control is a continuous variable; task timing is a dummy variable (1 = near future, 2 = distant future); and task focus is a dummy variable (0 = enjoyment focus, 1 = effort focus). Entries in the table represent unstandardized coefficients. Standard errors are reported in parentheses.

* p < 0.05,

** *p < 0.001*.

As expected, we found a significant three-way interaction between task timing, self-control, and task focus, *b* = −1.55; *t*_(188)_ = −2.39, *p* = 0.0176. Decomposing the three-way interaction revealed that the interaction between task timing and self-control was significant among participants in the effort-focused condition, *b* = −1.34; *t*_(188)_ = −3.43, *p* = 0.0007, further reinforcing the results of experiments 1 and 2. LSC participants reported higher self-efficacy for the distant-future task than for the near-future task, *b* = 1.099; *t*_(188)_ = 3.18, *p* = 0.0017, whereas HSC participants reported marginally higher self-efficacy for the near-future task, *b* = −0.72; *t*_(188)_ = −1.82, *p* = 0.071. When the task description highlighted the enjoyable aspects of the task (enjoyable-focused condition), the interaction effect of task timing and self-control on self-efficacy was not significant, *b* = 0.52; *t*_(188)_ = 0.41, *p* = 0.68.

#### Task engagement

An additional regression analysis on participants' willingness to sign up for the vacation also revealed a significant three-way interaction between task timing, self-control, and task focus, *b* = −1.62; *t*_(188)_ = −3.94, *p* = 0.004; The complete analysis is provided in Table 4, column 2. The interaction between task timing and self-control was significant among participants in the effort-focused condition, *b* = −1.27; *t*_(188)_ = −3.84, *p* = 0.0002; specifically, LSC participants were more willing to engage in the distant-future task than in the near-future task, *b* = 0.91; *t*_(188)_ = 3.09, *p* = 0.002, whereas HSC participants were more willing to engage in the near-future task than in the distant future task, *b* = −82; *t*_(188)_ = −2.44, *p* = 0.015. In the enjoyable-focused condition, the interaction was not significant, *b* = 0.35; *t*_(188)_ = 0.79, *p* = 0.42.

#### Underlying process

**Figure 4:**
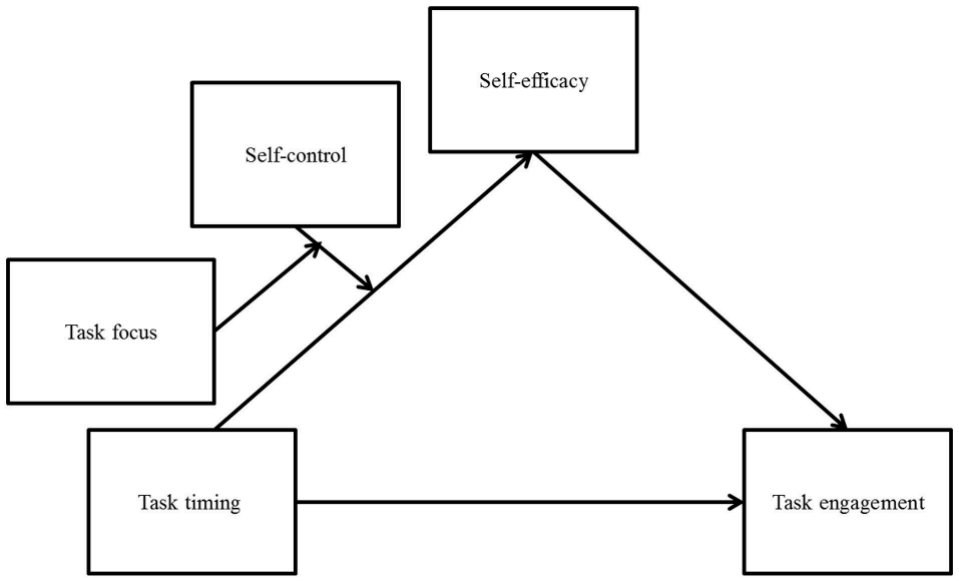
Self-efficacy mediates the effect of task timing (near future or distant future) on task engagement dependent upon task focus (effortful focus or enjoyable focus) for different levels of self-control.

To examine the mediating role of self-efficacy, we used the PROCESS macro based on Hayes's Model 11 with 5,000 bootstrap samples (Hayes, 2013; Figure 4 presents a schematic presentation of the model). As hypothesized, self-efficacy mediated the effect of task timing on willingness to plan the vacation *only* for participants in the effort-focused condition; this was true both for LSC participants (*b* = 0.28, *SE* = 0.14, CI 95%: 0.07–0.62) and for HSC participants (*b* = −0.18, *SE* = 0.11, CI 95%: −0.44 to −0.001).

The results of experiment 3 suggest that the effects observed in Studies 1 and 2 are replicated when the effortful aspects of the task are emphasized, even if the task is associated with something pleasant, like a vacation. Thus, in the effort-focused condition, LSC participants were more willing to engage in the distant-future task, whereas HSC participants were more willing to engage in the near-future task. These intentions were mediated by self-efficacy. Notably, when enjoyable aspects of the same task were highlighted, these effects were attenuated.

## General discussion

Across three experiments, we show that individuals with LSC have higher SSE when an effortful task is in the distant future than when it is in the near future. Therefore, they are more willing to engage in the former than in the latter. In contrast, individuals with HSC have higher SSE with regard to near future tasks, and are therefore willing to engage in these tasks over distant future tasks. We observed these effects in cases where the effortful aspect of the task was salient, whereas the effects were eliminated when participant focus shifted to the enjoyable aspects of the task.

These findings have important theoretical implications for our understanding of self-efficacy, as well as of self-control. Specifically, within the literature on self-efficacy: First, we show that individuals experience different levels of task self-efficacy at different points in time, and that these differences depend on their dispositional self-control. Second, we show that even tasks with enjoyable associations, such as planning a vacation, are more susceptible to differences in self-efficacy when framed as demanding effort. In other words, the influence of task timing and self-control on self-efficacy can be triggered by task framing, and thus may account for differences in motivation to engage in almost any task. Within the literature on self-control: First, our findings may we offer a new interpretation for LSC individuals behavior. While theories of self-control all suggest that LSC individuals' behavior reflects self-control failure in dilemmas [vice vs. virtue (Wertenbroch, 1998), want vs. should (Bazerman et al., 1998), desire vs. willpower (Hoch and Loewenstein, 1991), heart vs. mind (Shiv and Fedorikhin, 1999), and do wrong vs. do right (de Ridder et al., 2011; Ein-Gar and Sagiv, 2014)]. We show that there are cases in which LSC individuals may express willingness to engage in effortful tasks if their self-efficacy is high. This may imply that, sometimes, LSC individuals procrastinate, not because they fail at self-control, but because they experience higher self-efficacy in the future as compared to the present. Second, individuals with HSC have experienced success in such tasks in the past; therefore, they presumably hold stable self-efficacy perceptions toward future effortful tasks. We show that even HSC individuals experience different self-efficacy levels at different points in time, and, as a result, they express different degrees of eagerness to engage in effortful tasks, important as they may be.

Given that the current research introduces the role of self-efficacy in driving HSC and LSC individuals to perform effortful tasks at different points in time, it opens a few potential research avenues for exploring processes that elicit self-efficacy perceptions. For example, Ein-Gar (2015) suggests that HSC and LSC individuals respond differently to future commitments because the distant future is vague and abstract while the present is more concrete. Future research may explore whether LSC individuals, who express an optimism bias, interpret this vagueness as an advantage, believing they would be able to work around their schedule to find the time to uphold their commitment, leading to higher task efficacy assessment. HSC individuals, however, who express a pessimism bias, may interpret this vagueness as a disadvantage, such that they cannot be certain they would be able to uphold their commitment, and therefore they feel lower task efficacy. Another avenue could explore whether differences in self-efficacy are dependent upon information availability. It could be that efficacy is not influenced by the abstractness or concreteness of the task timing, but rather by how much information is accessible about the task's demands. As such, HSC individuals may be more willing to engage in a task the more information they have about it. Information accessibility can lead to feeling power over the situation, allowing them to be better prepared for it, which could lead to high task efficacy and higher engagement intentions. LSC individuals, however, may be intimidated by excessive information because it signals task complexity or difficulty, and therefore might experience low task efficacy. Hence, LSC individuals may be less likely to engage in a task when more information is accessible.

Our findings elucidate the distinction between future orientation and future preference. A future orientation refers to a tendency to think more about the future as compared with the present and is associated with more goal driven and self-controlled behavior (Strathman et al., 1994; Zimbardo et al., 1997; Zimbardo and Boyd, 1999). However, a future orientation does not necessarily equate to a future preference. In this research, we show that individuals with HSC demonstrate a higher likelihood to engage in an effortful task in the near future than the distant future, regardless of whether or not they might have a tendency to focus on the distant-future in time orientation (Ein-Gar et al., 2012). Future research can further explore whether or not time orientation has a stronger role in activating the motivation to pursue a goal, while time preference has a stronger role in activating perceptions regarding one's abilities to attain the goal.

This research is not without limitations. First, we measured pre-engagement declarations of intentions, but not the actual performances of the tasks. Such declarations of intent serve as a “foot in the door,” and are valid predictors of subsequent behavior (Ajzen and Madden, 1986; Maddux et al., 1986; Fishbein and Ajzen, 2009). Nonetheless, future research should reexamine the relationships studied here while taking into account possible conditions under which pre-engagement declarations are more or less likely to accurately predict actual engagement. For instance, individuals with LSC who expect to have high self-efficacy for effortful tasks in the distant future, and therefore commit themselves to these tasks, may eventually end up not being able to complete these effortful tasks on time. This vicious circle for LSC individuals could lead to more failures and, in turn, a greater sense of being low in self-control. However, if we provide such individuals with mechanisms to persist throughout their engagement in such tasks we might break this circle.

Second, our theoretical model focused on dispositional self-control, leaving room to test the applicability of the model for state self-control. We suggest and show that individuals with dispositional differences in self-control interpret the future tasks differently. State self-control, on the other hand, reflects an individual's actual state of resources, such that individuals are in an ego-depleted state after performing a depleting task and may not have sufficient resources to adequately complete subsequent tasks that are demanding of self-control (Baumeister et al., 1998; Hagger et al., 2010). Future research may explore whether non-depleted individuals will show the same crossover effect found in this study, where individual engagement in the task depends upon their dispositional self-control level. However, depleted individuals are not expected to express willingness to engage in an effortful task regardless of its timing, whether it is because they lack the resources to process the decision at present, or because they are unable to anticipate their future resources. In other words, resource depletion may diminish the crossover effect we report in our studies.

The present research has shown that the effortful aspects of a task play a crucial role in individual willingness to engage in that task. The results of this study suggest that policymakers, managers, and educators can emphasize particular aspects of a task along with the task's time specifications to shape an individual's self-efficacy in accordance with their levels of self-control and ultimately encourage them to engage in effortful tasks.

## Author contributions

DE and YS developed the experimental concept of the research. DE designed and collected the data for the third study. DE and YS designed and collected the data for the first and second studies. The studies were conducted from May 2014 to July 2015. Both authors jointly analyzed the data and approved the final version of the submitted manuscript.

### Conflict of interest statement

The authors declare that the research was conducted in the absence of any commercial or financial relationships that could be construed as a potential conflict of interest.
