# Supplementary material for: Self-control and Task Timing Shift Self-efficacy and Influence Willingness to Engage in Effortful Tasks
Source: Front Psychol. 2017 Oct 11;8:1788. doi: 10.3389/fpsyg.2017.01788 (PMC5641896; doi:10.3389/fpsyg.2017.01788)
Supplement: Supplementary file 1 [file DataSheet1.pdf]

Appendix A: Task Descriptions

Experiment 1: Willingness to Join a Financial Coaching Program

A well-known financial institution, with numerous branches dispersed widely throughout the country, is launching a social marketing campaign directed at current or potential clients. This campaign includes individual counseling and coaching services to encourage smart and cost-effective management of one's finances—services that are offered free of charge.

As part of the campaign, the bank's employees will devote several hours per week to building a personal plan for each client (current or potential) in accordance with his or her individual needs. The client can decide how many meetings to attend and which goals he or she would like to achieve in those meetings (for example, receiving instruction on effective management of a household budget, managing an investment portfolio, and learning to use software to track expenses).

To this end, the financial institution has trained its employees to provide courteous and appropriate service to clients who are interested in these services. In addition, the financial institution has allocated its employees' work hours, in various branches nationwide and at various times, to enable employees to be available to meet personally with interested clients.

It is important to note that these meetings take place free of charge for current or potential clients. Each personal meeting provides current clients with a discount on bank fees, and the more meetings the client attends, the greater the discount he or she will receive. A client who cannot attend a scheduled meeting must cancel the meeting at least 24 hours in advance.

In order to receive personal coaching, the client must prepare relevant documents, including pay stubs, information on day-to-day expenses, and goals that he or she would like to achieve in the near future.

Experiment 2: Willingness to Join a Running/Walking Group

“Co-on-the-Go” is a new company that organizes “running/walking groups” with professional trainers near your place of residence.

A “running/walking group” is a group of members (between 4 and 12 members) who meet a few times a week with a trainer who walks/runs with the group. During the activity, the trainer provides useful comments on how to better walk/run, teaches members to keep track of their exercise, and builds a smart, adjustable, and gradual exercise program.

The advantage of joining such a group is obvious: You benefit from having a professional trainer follow you closely, and you get to be part of a social group, which provides emotional support and makes the exercise fun!

As part of the company’s effort to penetrate the market, “Co-on-the-Go” is offering participants in this survey a special rate: Participants who pre-register to try out this program will pay $5.00 (instead of $10.00) per hour. A personal trainer costs $30.00–$60.00 per hour; this program allows you to enjoy the expertise of a personal trainer for the price of $5.00 per hour.

When registering for the program, each member is assigned to a group depending on demographic preferences (age and gender), physical state (walking group or running group), number of weekly sessions (2, 3, or 4) and time preferences (mornings or evenings).

Experiment 3: Planning a Vacation

Imagine that you have received a monetary bonus at work. Using this bonus together with some money you have put aside, you want to go with your spouse on a long vacation. During this vacation, you will visit a few places, stay at hotels, and meet old friends and family you have not seen for a long time. You expect that arranging this vacation will demand some time and effort./You expect that arranging this vacation will be very pleasant and fun.

Assume that you plan to travel sometime during July–August. Your travel agent has found a great deal for your main flight.

The deal ends in **February,** which means that you will need to make all the decisions and preparations by **January**./The deal ends in **July**, which means that you will need to make all the decisions and preparations by **June**.
